# A Large Repertoire of Parasite Epitopes Matched by a Large Repertoire of Host Immune Receptors in an Invertebrate Host/Parasite Model

**DOI:** 10.1371/journal.pntd.0000813

**Published:** 2010-09-07

**Authors:** Yves Moné, Benjamin Gourbal, David Duval, Louis Du Pasquier, Sylvie Kieffer-Jaquinod, Guillaume Mitta

**Affiliations:** 1 Parasitologie Fonctionnelle et Evolutive, UMR 5244, CNRS Université de Perpignan, Perpignan, France; 2 University of Basel, Institute of Zoology and Evolutionary Biology, Basel, Switzerland; 3 Laboratoire d'étude de la dynamique des protéomes CEA-DSV/IRTSV, Grenoble, France; Biomedical Research Institute, United States of America

## Abstract

For many decades, invertebrate immunity was believed to be non-adaptive, poorly specific, relying exclusively on sometimes multiple but germ-line encoded innate receptors and effectors. But recent studies performed in different invertebrate species have shaken this paradigm by providing evidence for various types of somatic adaptations at the level of putative immune receptors leading to an enlarged repertoire of recognition molecules. Fibrinogen Related Proteins (FREPs) from the mollusc *Biomphalaria glabrata* are an example of these putative immune receptors. They are known to be involved in reactions against trematode parasites. Following not yet well understood somatic mechanisms, the FREP repertoire varies considerably from one snail to another, showing a trend towards an individualization of the putative immune repertoire almost comparable to that described from vertebrate adaptive immune system. Nevertheless, their antigenic targets remain unknown. In this study, we show that a specific set of these highly variable FREPs from *B. glabrata* forms complexes with similarly highly polymorphic and individually variable mucin molecules from its specific trematode parasite *S. mansoni* (*Schistosoma mansoni* Polymorphic Mucins: *Sm*PoMucs). This is the first evidence of the interaction between diversified immune receptors and antigenic variant in an invertebrate host/pathogen model. The same order of magnitude in the diversity of the parasite epitopes and the one of the FREP suggests co-evolutionary dynamics between host and parasite regarding this set of determinants that could explain population features like the compatibility polymorphism observed in *B. glabrata*/*S. mansoni* interaction. In addition, we identified a third partner associated with the FREPs/*Sm*PoMucs in the immune complex: a Thioester containing Protein (TEP) belonging to a molecular category that plays a role in phagocytosis or encapsulation following recognition. The presence of this last partner in this immune complex argues in favor of the involvement of the formed complex in parasite recognition and elimination from the host.

## Author Summary

Contrary to the traditional view that immunity in invertebrates is limited to innate mechanisms, recent studies have shown that these several species of protostome invertebrates express putative immune receptors that can be somatically diversified in a way resulting in an analogy with Immunoglobulins or T Cell Receptors of vertebrate species. Other studies have shown the existence of putative antigenic variant counterparts in their specific parasite, as would be expected in an “arms race” between both protagonists. However, the interaction between these immune receptors and antigens was never demonstrated in an interaction involving an invertebrate and its specific pathogen. We demonstrate such an interaction in the present study. We show that a specific set of highly variable immune receptors of the mollusc *Biomphalaria glabrata* forms immune complexes with highly polymorphic and individually variable mucin determinants from its specific trematode parasite *S. mansoni*. We demonstrate for the first time in an invertebrate host-parasite interaction that a large repertoire of parasite epitopes matched a large repertoire of host immune receptors.

## Introduction

Understanding host-parasite interactions represents a major challenge in evolutionary biology. Parasites cause substantial deleterious effects on their hosts, and therefore represent a major driving force for their evolution [1]. In parallel, parasites have to cope with the evolving host-defence mechanisms, i.e. they must co-evolve with their host to avoid elimination. This antagonistic co-evolution in host-parasite interactions can be illustrated by an arms race in which both host and parasite develop mechanisms to circumvent weapons developed by their opponent. In this context, evolutionary hypotheses like the Red Queen Hypothesis [2] predict that diversity and polymorphism of molecules occurs especially on molecules that play key roles in the host-parasite interplay [3].

In vertebrate host/parasite interactions, adaptive immunity is the ultimate outcome of this molecular arms race. Vertebrates possess an extraordinary system able to generate somatically an exceptional diversity of antigen-specific receptors [4], [5], [6]. It consists in a ‘do-it-yourself kit’, i.e a set of gene segments to be assembled during the ontogeny of lymphocyte that randomly generates receptors. This adaptive immune system can recognize and initiate a protective response against most of the pathogen/antigen encountered. Indeed, gnathostomes as well as agnathes, seem to be able to generate a highly diverse repertoire of lymphocytes, each bearing a different cell surface antigen receptor [7], [8]. The interaction of the lymphocyte receptor with the epitope present on the antigen leads to a signal transduction and eventually to an effector phase leading to the neutralization or the destruction of the antigen. These diversified immune receptors can be different between vertebrate lineages. They are members of the immunoglobulin superfamily for B or T Cell Receptors in gnathostomes and members of the leucine rich repeat family or Variable Lymphocyte Receptors in agnathans, but in all cases they are generated through recombinatorial processes occurring somatically during lymphocyte differentiation and proliferation. The convergent evolution in all vertebrates of these different genes leading to the acquisition of a vast repertoire of somatically generated receptors proves the high selective value of this mechanism in the living kingdom and suggests that it might be found elsewhere. For the pathogen counterparts, a variety of mechanisms permitting evasion of the host's immune response exist in pathogenic bacteria and viruses [9]. But as expected in an arms race perspective, diversity, polymorphism and variation of antigens from pathogen is a widespread strategy also described (i) from numerous pathogens belonging to distant evolutionary lineages [10] and (ii) for most of the eukaryotic parasites [11].

In the case of invertebrate hosts and their parasites, the picture was believed to be completely different since the prevailing view was that invertebrates have no acquired adaptive immunity, and their immune system being innate would exhibit less diversity of the receptor repertoire and hence less specificity. The detection of parasites by these organisms was believed to rely exclusively on invariable germline-encoded immune receptors that recognize microbial antigens to limit pathogen invasion [12]. Recent studies have somehow shaken this paradigm. They report the existence of polymorphic and diversified putative immune receptor sequences that are somatically generated, that varies considerably from one individual to the other and that leads to an enlarged repertoire of putative recognition molecules. This was reported in echinoderms (sea urchin; [13]), insects (*Drosophila melanogaster and Anopheles gambiae*; [14], [15]), crustaceans [16] and molluscs (*Biomphalaria glabrata*; [17]). These studies have suggested the existence of a form of specific adaptative immunity in several invertebrates, without providing mechanism, which raised some doubts in the mind of traditional immunologists (for the polemic see [18], [19]). In addition, the direct proof of a role in immunity of these molecules is not provided. Do these diversified molecules actually interact with antigens? Are they able to interact with antigenic variants from parasites that are expected, in an arms race perspective, to be diversified and/or polymorphic? We propose to address this last crucial question in the present study.

As a model we choose the interaction between the trematode *Schistosoma mansoni* and its mollusc host *Biomphalaria glabrata*, in which several pieces of exciting data were obtained. Firstly, incubations of *B. glabrata* plasma extracts and soluble antigens from trematodes led to the formation of molecular complexes [20], [21]. *B. glabrata* molecules involved in these complexes were characterized, they were called FREPs for Fibrinogen Related Proteins [21]. The *FREP* genes belong to a multigene family of at least fourteen members [22], [23]. FREPs consist of one or two amino-terminal IgSF domains and a carboxyl-terminal fibrinogen domain. These molecules undergo apparently somatic variations leading to a remarkable diversification [17]. The superimposition of allelic polymorphism and somatic processes can lead to the expression of 45 isoforms of FREP3 per individual [17]. These genes encode lectin-like hemolymph polypeptides that are able to bind to *E. paraensei* sporocysts and a variety of microbes [24]. However the ligands themselves are still mysterious. FREP expression increases in response to challenge with the trematode parasites, *Echinostoma paraensei* and *Schistosoma mansoni* [21], [25]. In the parasite *S. mansoni*, we identified recently polymorphic mucins [26]. They were called *Sm*PoMuc (for *S. mansoni* Polymorphic Mucins). They display a high level of intra- and inter-strain polymorphism due to a complex hierarchical system that efficiently generates polymorphic variants based on a relatively low number of genes [27]. We hypothesise that these mucins could contain the epitopes that interact with the immune receptors from *B. glabrata* and make the hypothesis that FREPs are among those receptors.

To test this hypothesis we developed two assays. Firstly, we developed a global proteomic approach to the interactome between parasite extracts and plasma extracts from the mollusc host. Co-incubation and precipitation of this total extract led to the identification of *Sm*PoMucs and FREPs in the same fraction. Secondly, the direct interaction of these two partners was confirmed by Co-Immunoprecipitation experiments using antibodies raised specifically against *Sm*PoMuc. Another interesting partner was coimmunoprecipitated in the same molecular complex. It corresponds to a putative opsonin, the ThioEster-containing Protein from *B. glabrata*.

## Materials and Methods

### Accession numbers

Nucleotide sequence data reported in this paper are available in the GenBank database under the accession numbers: HM003905 to HM003908, HM038098 to HM038105 and HM237113 to HM237135.

### Host and parasite strains and protein sample preparation

#### Ethics statement

Our laboratory has received the permit N° A 66040 for experiments on animals from both French Ministère de l'Agriculture et de la Pêche and French Ministère de l'Education Nationale de la Recherche et de la Technologie. Housing, breeding and animal care of the mice followed the ethical requirements of our country. The experimenter possesses the official certificate for animal experimentation delivered by both ministries (Décret n° 87–848 du 19 octobre 1987; number of the authorization 007083).

#### Parasite and host breeding and *in-vitro* culture procedures

Two strains of *S. mansoni* were used in this study, a Brazilian strain and a Guadeloupean strain the first of which is compatible (C strain) and the second of which is incompatible (IC strain) with a single Brazilian mollusc strain [28]. Each strain was maintained in (i) their sympatric strain of *B. glabrata* and in (ii) hamsters (*Mesocricetus auratus*) as described previously [28]. Miracidia from *S. mansoni* C and IC were hatched from eggs axenically recovered from 60-days infected hamster livers, according to the previously described procedure [26]. Briefly, livers were collected and kept overnight at 4°C in sterile saline solution (NaCl 150 mM), containing an antibiotic/antimycotic mixture (penicillin 100 units/ml, streptomycin 0.1 mg/ml, amphotericin B 0.25 µg/ml; Sigma). The livers were then homogenized and the eggs were filtered and washed. Miracidia were hatched from eggs in sterile water. Miracidia were recovered by pipetting and concentrated by sedimentation on ice for 1-h and directly submitted to *in vitro* transformation to obtain primary sporocysts (Sp1) [29]. Miracidia were cultured at 26°C in sterile Chernin's balanced salt solution (CBSS, [30]) containing the antibiotic/antimycotic mixture previously described [31]. Full transformation of miracidia to Sp1 occurred within 24 hours. Sporocysts were spun down (600 g for 5 min) and frozen at −80°C.

#### Native extraction of sporocysts

For each strain, 40,000 sporocysts were resuspended in 200µl TBS containing tween 20 (0.05%, v/v) and antiprotease cocktail (complete protease inhibitor cocktail, Roche). Then, they were submitted to sonication (Vibracell 75185 apparatus, 4 pulses of 20 seconds at 20% of amplitude on ice). Twenty µl of glass beads were added and the sample was vortexed (2700 rpm; 30 min; 4°C) and centrifuged (6000g; 30 min; 4°C). The supernatant was recovered and conserved at −80°C. The total protein amount present in the final sporocyst sample was determined with 2-D Quant Kit (Amersham Biosciences).

#### Plasma protein recovery

Hemolymph of two hundred Brazilian *B. glabrata* snails (BgBRA) (9–13 mm) was extracted as previously described [32]. It represents a total volume of 20ml approximately. A centrifugation (3000g; 10min; 4°C) was performed to pellet hemocytes and the plasma recovered (supernatant). Then, haemoglobin was removed from plasma using an ultra-centrifugation procedure (55 000 rpm; 2.5 hours; 4°C). Quantification of total protein concentration was performed with the 2-D Quant Kit (Amersham Bioscience). Plasmas were conserved at −80°C.

### S. mansoni/B. glabrata interactome experiments

Fifty µg of sporocyst extracts from C or IC strain and 750µg of plasma extracts were used for each interactome experiment. After thawing, extracts were submitted to a centrifugation step of 7 500g for 30 min at 4°C. The supernatants were recovered, mixed and incubated at 26°C for 2.5 hours. After incubation precipitated materials were recovered by two successive centrifugation steps at 7 500g and 15 000g for 30 min and at 4°C. The same procedure was realised with sporocyst and plasma extracts alone to identify proteins precipitating spontaneously. Precipitated proteins were resuspended in 7µl of UTCD (8M urea, 40 mM TRIS, 4% CHAPS, 60 mM DTT), 3µl of laemmli buffer 3× was added and precipitates were analysed by SDS-PAGE. Gels were silver stained using a staining procedure compatible with mass spectrometry analysis [33].

### Production and purification of recombinant SmPoMuc and co-immunoprecipitation

#### Construction of expression vector and production of recombinant *Sm*PoMuc1

The last 699 bp sequence of *Sm*PoMuc1 (GenBank accession number: EU042599) encoding the constant C-terminal region (from amino acid 199 to amino acid 432) was amplified and cloned into the NheI/SacI sites of the pET200/D-TOPO expression vector in frame with a hexahistidine tag (Invitrogen). Briefly, the 699 bp cDNA fragment of *Sm*PoMuc1 (r*Sm*PoMuc) was obtained using a standard amplification reaction with the following primers containing NheI or SacI restriction sites (5′ primer : CTA-CTA-CTA-gct-agc-GTT-CCA-GAA-CAT-TTG-AAA-ACG-A and 3′ primer ATT-ATT-ACA-gag-ctc-ATC-AGC-TGC-AAT-TGG-TTG-AAT-CTT). Transformation of the plasmid construct was done in TOP10 chemically competent *E. coli* cells (Invitrogen) and sequencing was performed using T7 forward and reverse primers to verify its open reading frame.

For production of r*Sm*PoMuc-tagged protein, plasmid construct was transformed into Bl21 (DE3) *E. coli* competent cells. Transformed bacteria were grown in LB broth medium with kanamycin (50µg/ml) at 28°C. For protein expression, induction was performed when OD600 culture reached 0.5 by addition of IPTG at 0.7 mM and maintained overnight at 16°C.

The expressed recombinant protein was purified by IMAC using a Ni-NTA column under native conditions as recommended by the manufacturer (Invitrogen). Briefly, BL21 *E. coli* cultures expressing r*Sm*PoMuc were lysed under 20 mM imidazole and sonicated (15 pulses of 20 seconds at 97% of amplitude) on ice. The lysate was then centrifuged at 3000 g for 15 min at 4°C. The supernatant was added to 0.75 ml of packed nickel-nitrilotriacetic acid (Ni-NTA) agarose resin. The supernatant/resin mixture was incubated at room temperature for 20 minutes under shaking. Ni-NTA resin was washed using 4 different pH and imidazole steps (pH 8.0/20mM; pH6.0/50mM; pH 5.5/20mM; pH 8.0/20mM). r*Sm*PoMuc bound to Ni-NTA resin was then eluted with 150 mM imidazole at pH 8.0. Eluted r*Sm*PoMuc was further purified by Fast Protein Liquid Chromatography (FPLC) gel filtration on Superose 10/300 GL column (GE Healthcare) and concentrated on Amicon Ultra-4 Centrifugal Filter Unit 10 K NMWL (Millipore).

The purified His6-tagged r*Sm*PoMuc was then used to raise the anti-*Sm*PoMuc polyclonal antibody.

#### Production and purification of polyclonal antibodies against SmPoMuc1

An anti-r*Sm*PoMuc1 specific rabbit polyclonal antibody was produced according to standard procedures (Genepep, France). Briefly, 150 ìg of purified r*Sm*PoMuc (1mg/ml) was mixed with an equal volume of Freund's complete adjuvant and injected into 2 New Zealand white rabbits. Before the first injection of purified recombinant protein, 5 ml of blood was used to derive the pre-immune serum from the same rabbits. Four boosts of 150 ìg of recombinant protein were performed every 2 weeks following the primary injection. One week after the last injection, antiserum of rabbit was collected. The sensivity and specificity of this antiserum were evaluated by enzyme-linked immunosorbent assay (ELISA) and western blot. The titer of the rabbit immune serum was closed to 1/35000 (ELISA). No signal was obtained by ELISA (dilution 1/30) and western blot (dilution 1/500) with the pre-immune serum. Antiserum and pre-immune serum were precipitated by saturated ammonium sulfate and then purified by Protein A affinity chromatography. The specificity of the purified antibodies were evaluated by enzyme-linked immunosorbent assay (ELISA) and western blot.

#### Co-immunoprecipitation

Co-immunoprecipitation was accomplished using an antibody-coupling gel to precipitate the bait protein (sporocyst *Sm*PoMuc) and co-immunoprecipitate the interacting prey proteins. Anti-r*Sm*PoMuc antibody was coupled to an amine-reactive gel (ProFound co-immunoprecipitation kit, Pierce) overnight using slow agitation at room temperature.

Two different experimental procedures were used to isolate the bait and prey protein. During the first procedure, native sporocyst protein extract (50 µg) of C or IC strain were incubated with mollusc plasma extract (250 µg) for 2.5 hours at 26°C under slow agitation. Afterwards, the mix was passed through the anti-r*Sm*PoMuc-Coupled Resin. In the second experimental approach, the bait protein (sporocyst *Sm*PoMucs) was immobilized to anti-r*Sm*PoMuc-Coupled Resin and used to capture its partner passing the snail plasma through the resin. Co-immunoprecipitated proteins were then eluted using IgG elution buffer (Pierce), lyophilised and re-suspended in Laemmli buffer. As controls, the same procedures were performed using sporocyst extracts and plasma alone.

The eluted proteins were separated on a 12% SDS-PAGE. Gels were stained with silver according to a method compatible with mass spectrometry [33] or submitted to western-blot to confirm the presence of *Sm*PoMucs. The procedure was described in a previous study [34]. Briefly, after gel transfer to nitrocellulose, membranes were blocked, probed with anti-r*Sm*PoMuc (1/1000 dilution) and revealed with horse radish peroxidase anti-rabbit IgG (1/5000 dilution) using SuperSignal West Pico Chemiluminescent Substrate kit (Pierce).

### Mass spectrometry analysis

The procedure used was previously described [26], [32], [35]. Bands containing the proteins of interest were excised from gels and digested with trypsin. Eluated peptides were lyophilised and analysed by mass spectrometry (EDyP Service laboratory, Grenoble, France). Peptides were analysed using a nanoscale capillary liquid chromatography Ultimate 3000 coupled to a LTQ-Orbitrap tandem mass spectrometer (nanoLC–MS/MS) (Mann M et al 2001; Ashton PD et al 2001). The resulting MS/MS spectra were processed and converted into peak lists in dta format using the SEQUEST algorithm for interrogation of protein or nucleotide sequence databases. Peptide masses were compared to virtual tryptic digestion of proteins from SwissProt-Trembl (other metazoan database) and to translated Expressed Sequences Tags database (dbEST) of *S.mansoni* (205 892 Ests) and *B.glabrata* (54 305 Ests) using Mascot (http://www.matrixscience.com/). No missed cleavages were allowed and some variable modifications were taken into account in the search such as Acetylation (Protein N-term), Oxidation and Dioxidation (M), and Trioxidation (C). Searches were performed using an error on experimental peptide mass values of ±15.0 ppm and an error for MS/MS fragment ion mass values of 1.0 Da.

Mascot results were validated using IRMa software (interpretation of Mascot results) developed by “EDyP Service” laboratory. IRMa avoids redundant proteins in the analysis and reduced false positive to less than 1%. A protein was considered to be correctly identified if at least two peptides were confidently matched with database sequences with a p-value<0.001 for each peptide. In addition, an overall Mascot score was given by the software to the identification, a score greater than 100 was considered significant (*p*<0.05, [36]).

### Cloning and sequencing of TEP and FREP2

The complete open reading frame (ORF) of BgTEP and FREP2 from our laboratory *B. glabrata* BRA strain were amplified using reverse transcription-polymerase chain reaction (RT-PCR). In order to investigate the variability of FREP2 sequences, total RNA was extracted individually from 5 snails (whole bodies) (9–13 mm). Concerning BgTEP, total RNA was extracted from a pool of five snails. Total RNA extractions from snails were performed using Trizol Reagent according to the manufacturer's instructions (Invitrogen).

Total RNA (2 µg) were reverse transcribed with oligo d(T)_17_ primers and Superscript II reverse transcriptase according to the manufacturer's instructions (Invitrogen). Two µl of the RT reaction was then used for PCR experiments with the following primers corresponding to:

- TEP cDNA (GenBank accession number : FJ480411). 5′ primer: ATG-AGA-ATG-AAG-CTG-AAT-TTG-ATT-TT; 3′ primer: CTA-TGG-GCA-ACA-GTT-GAG-GCA-AAC-ATC.

- FREP2 cDNA (GenBank accession number : AY012700). 5′ primer: ATG-GCG-TCG-CTA-CCA-CTT-CGA-CTT-GTT-C ; 3′ primer: TTA-GTT-TAG-CTC-TAT-TTC-TCT-AAT-TTT-C. The PCR was performed using Advantage 2 PCR Enzyme System (Clontech).The PCR products were amplified, purified and cloned into pCR4-TOPO vector according to the manufacturer's instructions (Invitrogen). Clones were then sequenced using GATC facilities (GATC Biotech, Germany). Thirty four sequences of FREP2 were analysed from the five separated individuals. Five clones were sequenced for BgTEP.

### Bioinformatic analysis

All sequence identified from databases or obtained in the present study were imported in the sequencer software (version 4.5). They were aligned and contiged. Primary structure analyses were performed using SignalP 3.0 to predict the presence of signal peptide, NetNglyc 1.0 and NetOGlyc 3.1 (http://www.cbs.dtu.dk/services/) to predict potential glycosylation sites. Putative proteolytic cleavage sites were predicted using PeptideCutter (http://www.expasy.ch/tools/peptidecutter/) program. Protein domain searches were performed using SMART (http://smart.embl-heidelberg.de/). An unrooted phylogenetic tree was constructed (based on the multiple alignment performed with ClustalW) using the neighbour-joining method with MEGA 4.0.2. [37]. The reliability of the tree was tested using a bootstrap test (1000 replicates). Recombinatorial events in BgBRA-FREP2 were investigated using Dna SP 5.10 software [38].

## Results

### An approach designed to identify immune complexes in S. mansoni/B. glabrata interaction

We incubated (i) extracts prepared from parasite sporocysts (intramolluscal stage of *S. mansoni*) and (ii) extracts from *B. glabrata* plasma known to contain Pattern Recognition Receptors like FREPs [21] and other lectins [39], [40]. We use sporocyts from two laboratory strains of *S. mansoni* (C and IC for Compatible and InCompatible, respectively) for these experiments. Both strains were chosen for this differential compatibility in the single host mollusc strain from Brazil. [26]. The C strain infects 100% of the molluscs when 10 miracidia per individual are used for infection. An average number of 3.6 sporocysts develop in the mollusc [28]. The IC strain infects only 4% of the molluscs using the same conditions.

**Figure 1:**
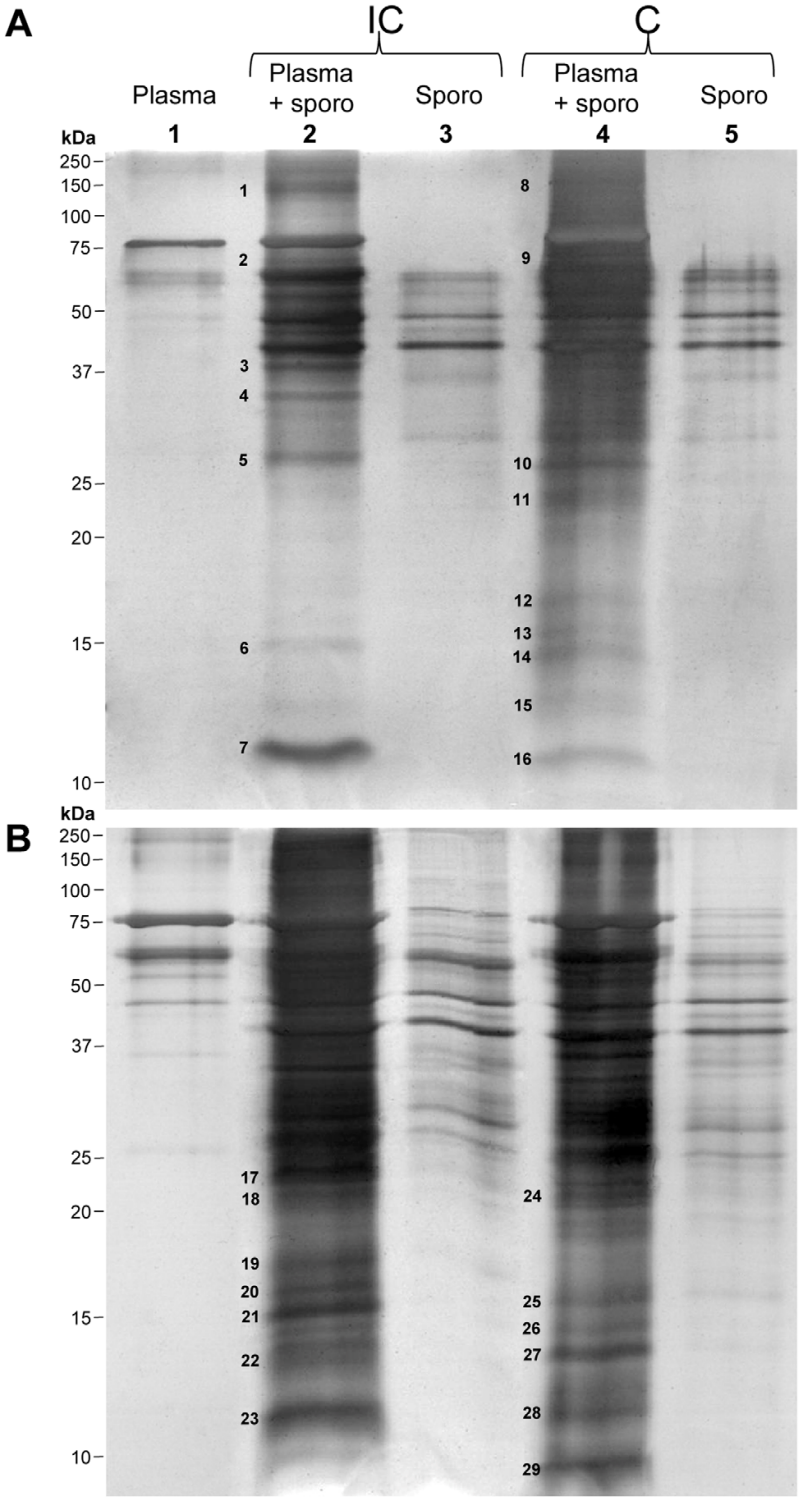
Interactome experiments. Precipitated proteins were pelleted using two centrifugation speeds and separated on 12.5% SDS-PAGE before silver staining. (**A**) 7 500g and (**B**) 15 000g. Compatible (C) or incompatible (IC) sporocyst (sporo) extracts and snail plasma were incubated together (lanes 2 and 4) or alone (controls, lanes 1, 3 and 5). Bands that differ between control and interactome experiments are numbered. These bands were cut, proteins submitted to tryptic digest and analysed by mass spectrometry for identification.

After incubation of host and parasite extracts, precipitated products were pelleted by centrifugation and analysed by SDS-PAGE. Different centrifugation speeds were used as well as different controls consisting in incubation and centrifugation of plasma or sporocyst extracts alone. The electrophoretic profiles of precipitate materials are shown in figure 1.

Gel analysis revealed that 29 bands were differentially represented between interaction experiments and controls (Figure 1). These bands were cut. The corresponding proteins were submitted to tryptic digest and analysed by tandem mass spectrometry for identification. Thirty proteins were identified - among them 20 are *S. mansoni* proteins (Table 1) and 10 are from *B. glabrata* (Table 2). During the experimental procedure, extracts were incubated 2.5 hours at 26°C. We cannot exclude the fact that proteolysis occurs. This phenomenon could explain why sometimes these multiple bands were obtained for the same proteins.

**Table 1:** *Schistosoma mansoni* interactome identification.

Function	Gel band no.	Protein ID	Accession no. (MSdb; *Sm*-dbEST)	Species	Strain	# of Peptides	Score
Glycoprotein	1-8	*Schistosoma mansoni* polymorphic mucin (*Sm*PoMuc)	A7UAX8_SCHMA, gi|166320028	*S.mansoni*	C/IC	6	340.44
	19	Secretory glycoprotein k5	Q2KMI8_SCHMA	*S.mansoni*	IC	5	214.28
	11-17-18-24	23 kDa integral membrane protein (Sm23) (tetraspanin)	gi|34654103, gi|34683177, gi|75967629	*S.mansoni*	C/IC	4	372.08
Calcium Binding Protein (EF-hand)	21-22-28	Egg Antigen SME 16	gi|166272806, gi|166281868, gi|166336526	*S.mansoni*	C/IC	6	524.34
	7-16-23-28-29	Tegument associated antigen	gi|34624832	*S.mansoni*	C/IC	5	272.88
	12-19	Antigen SM 20	gi|34726371	*S.mansoni*	C/IC	3	133.43
Chaperone Stress Protein	2-9	Heat shock protein HSP60	Q8MXA4_SCHMA, gi|75967703, gi|75968414, gi|34699546	*S.mansoni*	C/IC	18	1209.82
	10-11-14-18-22-27-28	Heat shock 70 kDa	gi|34662357, gi|34617312, gi|34732332, gi|34613005, gi|34627204, gi|34618891	*S.mansoni*	C/IC	13	940.55
	2-9	T-complex protein 1 subunit alpha	TCPA_SCHMA	*S.mansoni*	C/IC	10	714.09
	9	Heat shock protein 86	Q26582_SCHMA	*S.mansoni*	C	7	497.13
Anti-oxidant Enzyme	11-17-24	Thioredoxine peroxidase 3	gi|86548802, gi|5869153, gi|86551428	*S.mansoni*	C/IC	6	421.03
	10-24	Glutathione-S-transferase 26kDa	gi|34669417, gi|166288290	*S.mansoni*	C	5	322.44
	12-19-25	Gluthatione peroxidase	gi|34625624, gi|34610915, gi|166333339	*S.mansoni*	C/IC	4	284.83
	11-17-18-24	Thioredoxine peroxidase	gi|86548129, gi|86550734, gi|34624053	*S.mansoni*	C/IC	3	238.87
	23-29	Thioredoxin	gi|34670675	*S.mansoni*	C/IC	3	219.65
	5-10	Glutathione-S-transferase 28kDa	gi|166265034, gi|12353926	*S.mansoni*	C/IC	3	168.42
Immune Regulation Molecule	19-22-26-28	*Sm*SPO-1	gi|166340572	*S.mansoni*	C/IC	3	175.31
Other Function	10	Leucine rich repeat (LRR)	gi|86548857	*S.mansoni*	C	5	387.17
	1	major vault-like protein	gi|72291614	*S.mansoni*	IC	4	286.43
	26	GRIM-19-like protein	gi|86552247	*S.mansoni*	C	3	235.61

LC-MS/MS results were used to interrogate Swiss prot/Trembl database (MSdb) and *Schistosoma mansoni* ESTs database (dbEST). A protein was considered to be correctly identified if at least two peptides were confidently matched with a score greater than 100. ID: identified, C: compatible combination, IC: incompatible combination.

**Table 2:** *Biomphalaria glabrata* interactome identification.

Function	Gel band no.	Protein ID	Accession no. (MSdb; *Bg*-dbEST)	Species	Interaction with	# of Peptides	Score
Lectin	1-8	Fibrinogen Related Protein (FREP)	Q86GZ8_BIOGL, Q5YDA1_BIOGL, Q95UV9_BIOGL	*B.glabrata*	C/IC	8	349.5
	7-9-10-17-18-19-20-21-22-23-24-25-27-28-29	Galactose Binding lectin-like	gi|45596074, gi|163955927	*B.glabarata*	C/IC	9	666.28
Immune Relevant Molecules	28	Cystatin B-like	gi|84976026	*B.glabarata*	C	9	407.39
	2-4-9	Dec-1-like, Matrilin-like	gi|54425021, gi|146769285	*B.glabarata*	C/IC	7	425.37
	9-10-11-19-26	Aerolysin-like	gi|146770915, gi|157942185, gi|163956216	*B.glabarata*	C/IC	5	264.91
	27	Allograft inflamatory factor-like (AIF)	gi|149401339	*B.glabarata*	C	3	169.02
	23-29	Peroxinectin-like	gi|146765607	*B.glabarata*	C/IC	3	124.87
Other Function	3-10	Zinc metalloprotease-like	gi|141327900, gi|54424552	*B.glabarata*	C/IC	6	529.03
	22-28	Calcium binding protein 1	gi|163958069	*B.glabarata*	C/IC	4	274.76
	19-25	Stanniocalcin-like protein	gi|163956096	*B.glabarata*	C/IC	3	252.46

LC-MS/MS results were used to interrogate Swiss prot/Trembl database (MSdb) and *Biomphalaria glabrata* ESTs database (Bg-dbEST). A protein was considered to be correctly identified if at least two peptides were confidently matched with a score greater than 100. ID: identified, C: compatible combination, IC: incompatible combination.

*S. mansoni* proteins can be classified mainly into 5 groups taking into account their putative function and/or structural features: glycoproteins; calcium binding proteins; chaperone/stress proteins; antioxidant enzymes and proteins involved in immune regulation (Table 1). As far as *B. glabrata* proteins are concerned, they correspond mainly to lectins or other proteins listed in Table 2.

**Figure 2:**
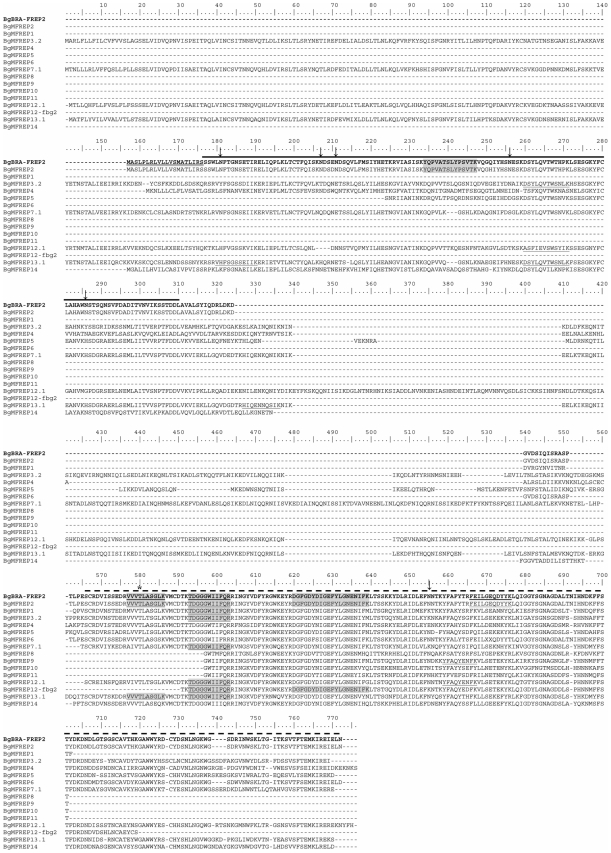
Alignment of BgBRA-FREP2 sequence with others FREPs from *B. glabrata*. Amino acid sequence of BgBRA-FREP2 (in bold, GenBank: HM003905) aligned with other FREPs family members from *B. glabrata*. Peptides identified by LC-MS/MS from the interactome approach and the coimmunoprecipitation approach are underlined and highlighted in grey, respectively. The putative signal peptide is double underlined, the putative N-glycosylation sites are indicated by arrows and the putative O-glycosylation site is indicated with an asterisk. The GenBank accession numbers of each entry are: BgMFREP1, AAK13549; BgMFREP2, AAK13550; BgMFREP3.2, AAK28656; BgMFREP4, AAK13551; BgMFREP5, AAK13546; BgMFREP6, AAK13552; BgMFREP7.1, AAK28657; BgMFREP8, AAK13553; BgMFREP9, AAK13554; BgMFREP10, AAK13555; BgMFREP11, AAK13556; BgMFREP12.1, AAO59918; BgMFREP12-fbg2, AAT58639; BgMFREP13.1, AAO59922; BgMFREP14, ABO61860. —— BgBRA-FREP2 IgSF domain; – – - BgBRA-FREP2 FBG domain.

The functions of the majority of the proteins identified are speculative because they are inferred from homologies with known molecules from other organisms after BLAST analysis and protein domain searches. Nevertheless, some of them are of particular interest in the present context, especially lectins from the host and glycoproteins from the parasite. Indeed host recognition molecules (like lectins) and carbohydrate containing molecular determinants from *S. mansoni* are excellent candidates for participating in an immune complexe. Several molecules belonging to these functional classes were identified. In *B. glabrata*, the FREPs [17], [21], and another putative lectin, a galactose binding-like, were clearly identified (Table 2). Different FREP family members were revealed using mass spectrometry. Among the peptides identified, some of them correspond specifically to FREP2, FREP12 and FREP13 (see Figure 2 for details).

**Figure 3:**
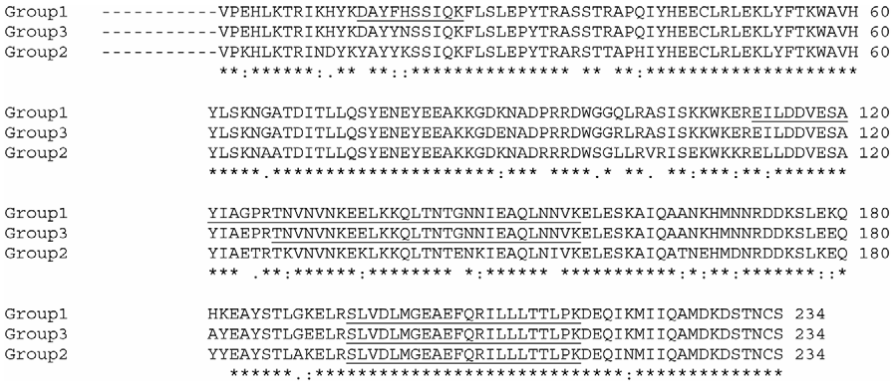
Amino acid sequences alignment of the C-terminal part of *Sm*PoMucs from the three identified groups. The peptides identified by LC-MSMS are underlined. Conserved positions are indicated by an asterisk. GenBank accession numbers: group 1 (EU042600), group 2 (EU042602) and group 3 (EU042633).

In *S. mansoni*, *Sm*PoMucs were precipitated (Table 1). As *Sm*PoMuc group 1-specific peptides were identified, the presence of the *Sm*PoMuc from the first group is affirmed (see Figure 3 for peptides identified). Nevertheless, we cannot exclude the presence of *Sm*PoMuc from the two other groups in the precipitated material (3 groups of *Sm*PoMucs were previously characterised see [34]. Other glycoproteins like the secretory glycoprotein K5 and the 23 kDa integral membrane protein (Sm23) from *S. mansoni* were also identified [41], [42].

Other proteins were identified that could be involved in protection of the parasite or in host immune response. Their putative role will be envisaged in the discussion.

### Coimmunoprecipitation: A Fibrinogen related protein (FREP 2) and a thioester-containing protein form a complex with SmPoMucs

We chose to focus then on the putative interaction between FREPs and *Sm*PoMucs. FREPs are highly variable molecules described in *B. glabrata*, and in at least four other genera of gastropods [21], [43] and related members, although with a different domain composition, exist in arthropods [44] and in cephalochordates [45]. All the observations on FREPs suggest that these molecules may act as highly diversified recognition and/or effector proteins somehow analogous to antibodies from vertebrate species [46], [47]. From an evolutionary point of view and in an arms race perspective, these diversified immune receptors are expected to interact with diversified antigens from the pathogen counterpart, but this remains to be demonstrated. *Sm*PoMucs identified in the present study represent possible ligands for these diversified host molecules. Indeed, these proteins correspond to polymorphic mucins that are secreted and preferentially expressed in miracidium or sporocyst stages [34]. *Sm*PoMucs are highly glycosylated and have an extraordinary level of polymorphism facing the diversified FREPs from *B. glabrata* that could represent a particularly well adapted set of immuno receptors or effectors.

To test this hypothesis and to determine which snail proteins may interact or form a complex with *Sm*PoMucs, we carried out CoImmunoPrecipitation (CoIP) experiments using antibodies raised against recombinant *Sm*PoMuc (r*Sm*PoMuc).

**Figure 4:**
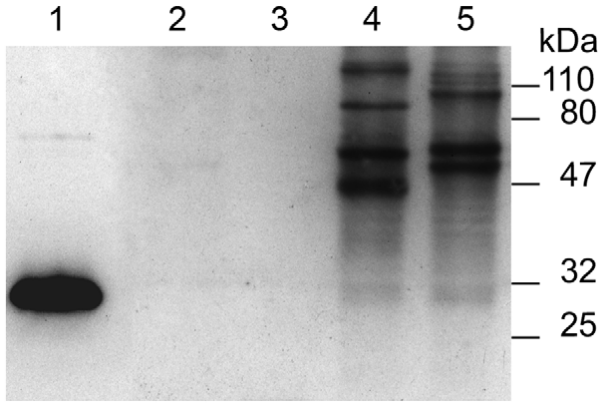
Anti-r*Sm*PoMuc antibodies specificity verified by Western blot. Lane 1: 30 ng of r*Sm*PoMuc ; Lane 2: 8 µg of *B. glabrata* whole extract ; Lane 3: 8 µg of *B. glabrata* plasma ; Lane 4: 8 µg of *S. mansoni* incompatible strain ; Lane 5: 8 µg of *S. mansoni* compatible strain. Extracts were separated by SDS-PAGE (12.5% gels), transferred on nitrocellulose membrane and probed with 1/1000 dilution of rabbit anti-r*Sm*PoMuc antibodies (purified IgG). Development was performed using horseradish peroxidase anti-rabbit IgG (1/5000 dilution) and chemiluminescent substrate.

Firstly, r*Sm*PoMuc corresponding to the C-terminal part of *Sm*PoMuc1 (234 last residues) was produced and purified to raise an anti-*Sm*PoMuc1 polyclonal antibody. After purification of IgG by protein A affinity chromatography, the sensivity and specificity of anti-*Sm*PoMuc1 antibody were evaluated by ELISA assay (data not shown) and western blot (Figure 4). In C and IC sporocyst extracts, only the bands corresponding to *Sm*PoMuc were revealed (Figure 4, lane 4 & 5). These profiles confirm the *Sm*PoMuc profile obtained in a previous study and show also that anti-*Sm*PoMuc1 polyclonal antibodies recognize all members of the *Sm*PoMuc family [34]. In addition, the absence of cross-reactivity with *B. glabrata* protein extracts was verified (Figure 4, lane 2 & 3). No signal was obtained in ELISA and Western blot assays using Protein A-purified IgG prepared from pre-immune serum (data not shown).

**Figure 5:**
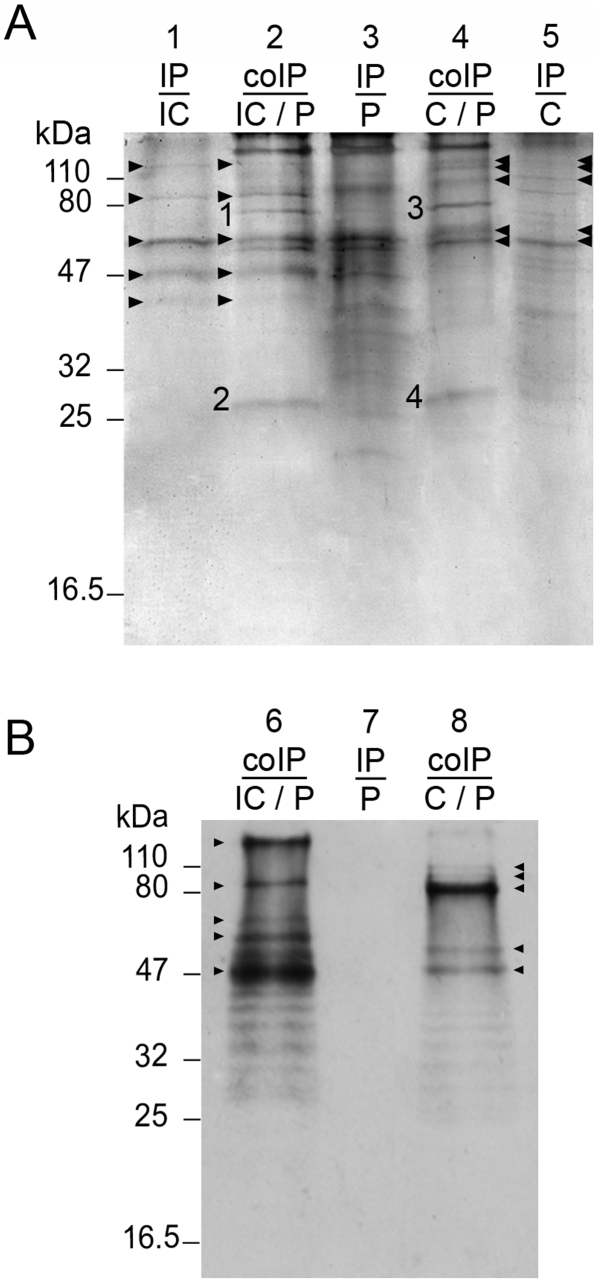
Immunoprecipitation and Coimmunoprecipitation experiments. **A.** CoImmunoPrecipitated (CoIP) and Immunoprecipitated (IP) extracts were separated by SDS-PAGE (12.5% gels) and silver-stained. Lanes 2 and 4 correspond to CoIP extracts. Lane 2: CoIP material obtained after incubation of sporocyst extracts from *S. mansoni* incompatible (IC) strain incubated with extracts from *B. glabrata* plasma (P). Lane 4: CoIP material obtained after incubation of sporocyst extracts from *S. mansoni* compatible (C) strain incubated with extracts from *B. glabrata* plasma (P). Lanes 1, 3 and 5 represent controls of Immunoprecipitated material. Lane 1: IP extracts from sporocyst of *S. mansoni* incompatible (IC) strain. Lane 3: IP extracts from *B. glabrata* plasma (P). Lane 5: IP extracts from sporocyst of *S. mansoni* Compatible (C) strain. **B.** Western-blot of immunoprecipitated (IP) and coImmunoPrecipitated (coIP) samples probed with anti-r*Sm*PoMuc antibody. Lane 6: CoIP material obtained after incubation of sporocyst extracts from *S. mansoni* incompatible (IC) strain incubated with extracts from *B. glabrata* plasma (P). Lane 7: IP extracts from *B. glabrata* plasma (P). Lane 8: CoIP material obtained after incubation of sporocyst extracts from *S. mansoni* compatible (C) strain incubated with extracts from *B. glabrata* plasma (P). Black arrow heads indicate the position of *Sm*PoMuc. Bands differentially represented between control and coIP samples are numbered (1 to 4). These four bands were cut and submitted to digestion and mass spectrometry analysis for identification.

For CoIP experiments, controls and coimmunoprecipitated extracts from C and IC combinations were separated by SDS-PAGE (Figure 5). The ability of antibodies to immunoprecipitate *Sm*PoMucs from C and IC sporocyst extracts was tested. The bands corresponding to *Sm*PoMucs are revealed by silver stain in immunoprecipitated sporocyst extracts (Figure 5A, lane 1 & 5). The identification of *Sm*PoMucs in coimmunoprecipitated samples was assayed by western blot (Figure 5B, lane 1 & 3) and confirmed by mass spectrometry. Bands corresponding to *Sm*PoMucs in coimmunoprecipitated extracts (Figure 5A, lane 2 & 4, position indicated by arrows) were cut, submitted to tryptic digest and analysed by liquid chromatography-tandem mass spectrometry (LC-MS/MS). These bands correspond to the different groups of *Sm*PoMucs as previously described (data not shown, [27]).

By comparison to controls, four specific bands were obtained for the coimmunoprecipitation assay (Figure 5 A; lane 2 bands n°1 and 2; lane 4 bands n°3 and 4). These bands were excised from the gel and submitted to mass spectrometry analysis. The same procedure was applied to the bands present at the same position in control snail plasma to ascertain protein identification after LC-MS/MS. Mass spectrometry analysis of the four bands of interest led to the identification of three proteins (Table 3). None of these proteins were identified for the corresponding bands in controls. As expected considering their position in the gel (∼70–75 kDa), bands 1 and 3 (from IC and C combinations, respectively) led to the same identifications: Fibrinogen-related proteins (FREPs) and a Thioester-containing protein (TEP), both from *B. glabrata*.

**Table 3:** Identification of coimmunoprecipitated proteins from *B. glabrata*.

Function	Gel band no.	Protein ID	Accession number		Species	Interaction with	# of Peptides	Score
			MSDB	dbEST				
Lectin	1-3	Fibrinogen Related Protein 2 (FREP 2)	Q95UV9_BIOGL	#	*B.glabrata*	C/IC	4	216.62
Immune Relevant Molecules	1-3	thioester-containing protein (BgTEP)	#	gi|149407840, gi|84976399, gi|157945681, gi|163957098	*B.glabrata*	C/IC	6	374.68
Other Function	2-4	alpha amylase like	#	gi|146763124, gi|163957465	*B.glabrata*	C/IC	7	473.53

LC-MS/MS results were used to interrogate Swiss prot/Trembl database (MSdb) and *Biomphalaria glabrata* ESTs database (*Bg*-dbEST).

A protein was considered to be correctly identified if at least two peptides were confidently matched with a score greater than 100.

ID: identified, C: compatible combination, IC: incompatible combination.

In the case of FREPs, 4 peptides were identified by LC-MS/MS analysis. These are contained in different FREP isoforms available in GenBank database (Figure 2). The identification of a FREP2-specific peptide (Figure 2) confirms that FREP2 is present in these two bands. However the presence of other FREP family members cannot be excluded.

Taking into account the variability previously observed in this gene family, we investigated FREP2 in our own mollusc strain from Brazil (BRA). The cDNA corresponding to FREP2 was amplified by RT-PCR using RNA extracted from seven *B. glabrata* BRA snails and specific oligonucleotides designed from FREP2 sequence available on databases (BgMFREP2, FREP2 from M line *B. glabrata*, GenBank Accession number: AY012700). The amplicons obtained were cloned. One clone was sequenced. This sequence was called BgBRA-FREP2 and deposited in GenBank (Accession number: HM003905). The overall sequence identity and similarity between BgMFREP2 and BgBRA-FREP2 (isoform 1, HM003905) are 99.2% and 99.7%, respectively. BgBRA-FREP2 shares the structure of BgMFREP2 which has already been described [48]. It contains one IgSF domain upstream the C-terminal fibrinogen domain (FBG) (Figure 2). In addition, we investigate the variability of FREP2 sequences. Using RT-PCR amplification, we amplified FREP2 from five individuals from the BRA strain. Then, we cloned the PCR product obtained for each individual and 12 clones were randomly picked and sequenced. As primers do not discriminate between FREP6 and FREP2, 26 and 34 sequences of these two FREPs were obtained respectively. BgBRA-FREP2 sequences were further analysed. As expected, these sequences display a high level of similarity (about 99%) at the nucleic acid level. Nevertheless, 23 of them are non redundant (GenBank accession numbers: HM237113 to HM237135), indicating a high degree of diversity (88%). Interestingly, two individuals express 7 and 8 different isoforms of FREP2, respectively while a maximum 3 loci per haplotype were estimated in a previous study [22]. No recombinatorial process was observed (using Dna SP 5.10 software) indicating that at least a part of this FREP2 diversity was generated by somatic nucleotide point mutations with a strong bias for transitions (A to G and T to C).

The four peptides identified by LC-MS/MS cover 14.28% of BgBRA-FREP2 (HM003905) deduced amino acid sequence (Figure 2). The theoretical molecular weight of BgBRA-FREP2 deduced amino acid sequence is 43.8 kDa. The observed molecular weight of BgBRA-FREP2 (Figure 5A), approximately 70kDa, is not in agreement with the theoretical molecular weight. This phenomenon could be explained by post-translational modifications. Indeed, FREPs are known to be heavily glycosylated proteins [21] and the electrophoretic migration profile of FREPs under reducing conditions were shown to be comprised between 40 and 75 kDa in a previous study [24]. In addition, seven putative glycosylation sites (6 N-linked glycosylation and 1 O-linked glycosylation sites) have been predicted in BgBRA-FREP2 using the NetNglyc 1.0 and NetOglyc 3.1 servers (http://www.cbs.dtu.dk/services/). Consequently, we hypothesize that this difference between theoretical and observed molecular weight is due to post-translational glycosylation events.

**Figure 6:**
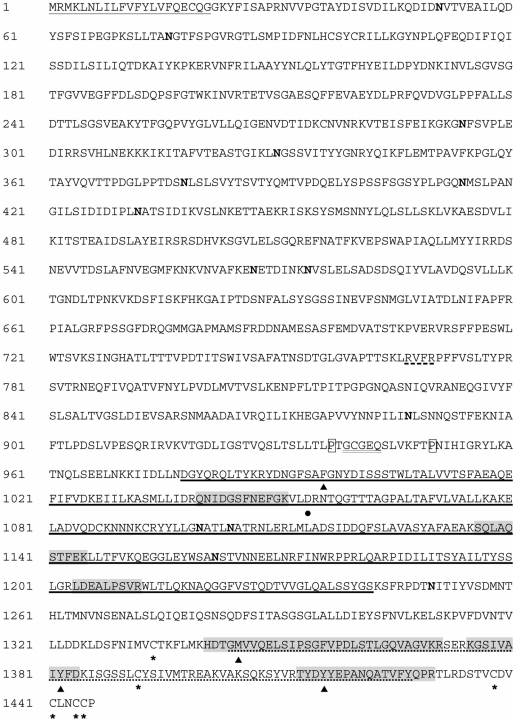
Deduced amino acid sequence of BgTEP. The complete coding sequence of BgBRATEP was obtained (GenBank: HM003907) and the deduced precursor sequence shown here. The 1446 amino acids of the precursor share the domains and motifs of the other known invertebrates TEP. Peptides, identified by LC-MS/MS, are highlighted in grey. The putative signal peptide is underlined, the putative N-glycosylation sites are indicated in bold. The thioester site is double underlined. Proline residues important for the thioester stability are boxed. • indicate the position of the aspartate residue (D1054) of the catalytic core. Cysteins belonging to the conserved cystein array shared by other TEPs are indicated by stars. ▴ indicate residues which form the protective hydrophobic/aromatic pocket of the thioester. The putative processing site for the cleavage is underlined with broken line. The Complement Component Domain is underlined and the Receptor-Binding Domain is dotted underlined.

Another protein was identified in the same bands 1 and 3. It corresponds to a thioester-containing protein (TEP) from *B. glabrata*. This TEP protein superfamily contains three different families of proteins which display distinct functions: (i) the vertebrate complement proteins (C3/C4/C5), (ii) the pan-protease inhibitors Alpha2 Macroglobulin (A2M) found in both vertebrates and invertebrates and finally, (iii) non classical A2M including TEPs subgroup only identified in invertebrate species and cell surface thioester containing protein isoforms (CD109 subgroup). We characterize the ORF of the *B. glabrata* TEP (BgTEP) from RNA of *B. glabrata* from the BRA strain (GenBank Accession Number HM003907). The deduced amino acid sequence corresponds to a precursor of 1446 amino acids. The peptides identified by LC-MS/MS cover 6.22% of the precursor sequence (Figure 6, Accession Number HM003907). The BgTEP sequences contain a putative 21 residue signal peptide as revealed by SignalP 3.0 analysis. It displays 14 putative N-glycosylation sites predicted by NetNGlyc 1.0 software. SMART program analysis reveals that BgTEPs contain the different domains shared by members of the TEP superfamily [49]. The canonical thioester motif (GCGEQ) of the TEP family is located from residue 939 to 943, and the thioester bond is likely to be formed between C940 and E942. Proline residues involved in the formation, stability and function of the thioester bond in the human C3 [50] are found around the thioester site. The four residues (F996, M1345, Y1382, Y1416) forming the hydrophobic/aromatic pocket for the protection of the thioester in the human C3 are also found at conserved position. The complement component and the Alpha2 Macroglobulin receptor binding domains are identified at amino acid positions 978–1242 (Protein domain ID: pfam PF07678) and 1343–1427 (Protein domain ID: pfam PF07677), respectively. BgTEP contains 13 cysteine residues, six of them are located at the C-terminus (1334–1445) forming a sequence signature shared with *Drosophila* TEPs, *Anopheles gambiae* aTEP-1, and *Chlamys farreri* TEP [51], [52], [53]. This last cysteine array is a specific signature of invertebrate TEPs [51], [52], [54] that is not shared by complement and A2M. Finally, BgTEPs possess an aspartate residue (D1054) replacing the catalytic histidine residue usually found in most of the protein of this family including invertebrate TEPs from *A. gambiae*, *A. aegypti*, *C. elegans*, *C. farreri* and *Ephaedusa tau* [52], [53]. This last feature is shared by a TEP from *Drosophila melanogaster* called TEP2. TEP2 was shown to be functional and required for the efficient phagocytosis of *E. coli* [55]. As the catalytic histidine residue determines the binding specificity of the thioester, this difference suggests an alternative binding mechanism already reported in other proteins of the family like alpha2 macroglobulin-related proteins [56].

Another interesting feature concerns the position of the protein in the gel. BgTEPs have a calculated molecular weight around 160 kDa which is not in agreement with the position of the protein in the gel (70kDa approximately). Interestingly, all the peptides identified by LC-MS/MS are located in the C-terminal part of the protein downstream the thioester site (Figure 6). These data suggest that we probably identified a cleaved C-terminal portion of the BgTEP. This suggests that BgTEP is processed like other members of the family. Indeed, human C3, alpha2 macroglobulins and *A. gambiae* TEP-1 have been shown to be activated by proteolysis [52]. However, no clear cut site has been identified in BgTEP, only a putative cleavage site sensitive to diverse proteases (trypsin, chymotrypsin, thermolysin, clostripain, LysC and LysN Lysyl endopeptidase, pepsin) has been predicted using PeptideCutter (http://www.expasy.ch/tools/peptidecutter/) program (see Figure 6).

**Figure 7:**
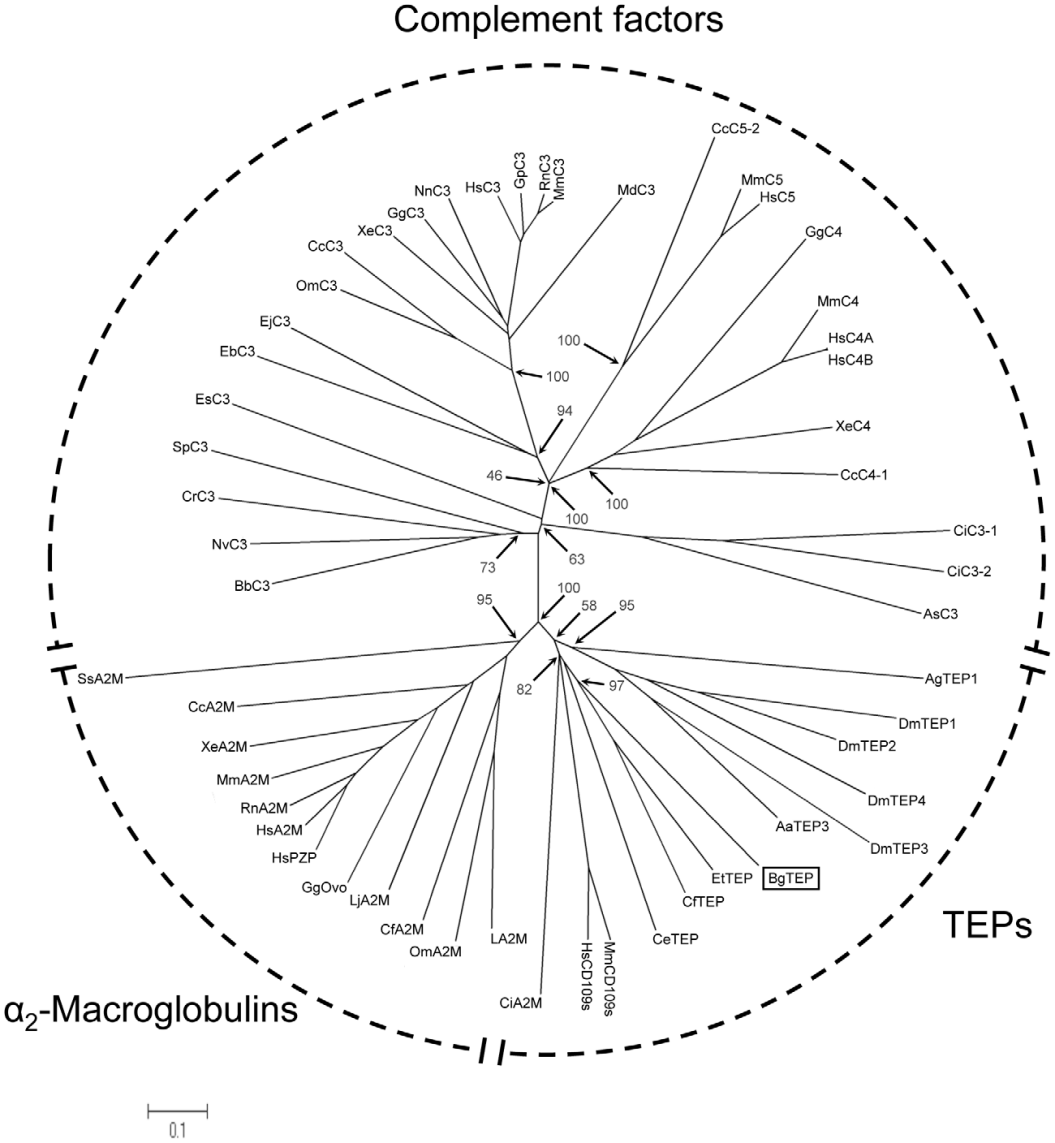
Phylogenetic position of BgTEP. The unrooted phylogenetic tree of thioester-containing proteins (54 sequences, cf. table 4) was produced by the neighbor-joining method based on the alignment of the sequences using CLUSTALW. Bootstrap values of 1000 replicates (%) are indicated for some nodes. The scale bar corresponds to 0.1 estimated amino-acid substitutions per site.

The phylogenetic position of BgTEP (Accession number: HM003907) was investigated in the present work. Phylogenetic analysis confirms the situation of BgTEP in the group of invertebrates TEPs. An unrooted phylogenetic tree was constructed with the neighbour-joining method using 54 sequences of TEPs (Figure 7 and Table 4). Three major groups can be distinguished in the TEP family: complement components group, the A2M group and the group formed by invertebrate TEPs and cell surface TEP (CD109). The topology obtained shows that A2M and invertebrate TEPs are more similar between them than they are with complement components. This phylogenetic distribution is consistent with those previously obtained for this protein family [49], [52], [53], [57]. BgTEP forms a cluster with other mollusc TEPs from *C. farreri* (39.5% similarity) and *E. tau* (55.1% similarity). This mollusc cluster forms a sister group of the insect TEPs from *A. gambiae* and *D. melanogaster*.

**Table 4:** Sequences of TEPs used to construct phylogenetic tree.

Code name	Name	Organism	Accession number (GenBank)
BgTEP	Snail TEP	*Biomphalaria glabrata*	HM003907
AgTEP1	Anopheles TEP-1	*Anopheles gambiae*	AAG00600
DmTEP1	Drosophila TEP1	*Drosophila melanogaster*	CAB87807
DmTEP2	Drosophila TEP2	*Drosophila melanogaster*	CAB87808
DmTEP3	Drosophila TEP3	*Drosophila melanogaster*	CAB87809
DmTEP4	Drosophila TEP4	*Drosophila melanogaster*	CAB87810
CeTEP	Caenorhabditis protein ZK337.1b	*Caenorhabditis elegans*	CAB05007
AaTEP3	Aedes TEP3	*Aedes aegypti*	EAT39604
EtTEP	Euphaedusa TEP	*Euphaedusa tau*	BAE44110
CfTEP	Zhikong scallop TEP	*Chlamys farreri*	ABP04060
BbC3	Amphioxus C3-like	*Branchiostoma belcheri*	BAB47146
NvC3	Starlet sea anemone C3	*Nematostella vectensis*	BAH22724
SeC3	Coral C3-like	*Swiftia exserta*	AAN86548
CrC3	Horseshoe crab C3	*Carcinoscorpius rotundicauda*	AAQ08323
EsC3	Bobtail squid C3	*Euprymna scolopes*	ACF04700
SpC3	Sea urchin C3	*Strongylocentrotus purpuratus*	AAC14396
CiC3-1	Ciona C3-1	*Ciona intestinalis*	Q8WPD8
CiC3-2	Ciona C3-2	*Ciona intestinalis*	Q8WPD7
AsC3	Ascidian C3	*Halocynthia rorezi*	BAA75069
EbC3	Hagfish C3	*Eptatretus burgeri*	CAA77677
EjC3	Lamprey C3	*Entosphenus japonicus*	Q00685
OmC3	Trout C3	*Oncorhynchus mykiss*	AAB05029
CcC3	Carp C3-H1	*Cyprinus carpio*	BAA36618
XeC3	Xenopus C3	*Xenopus laevis*	AAB60608
GgC3	Chicken C3	*Gallus gallus*	NP_990736
NnC3	Cobra C3	*Naja naja*	Q01833
HsC3	Human C3	*Homo sapiens*	P01024
GpC3	Guinea pig C3	*Cavia porcellus*	P12387
MdC3	Opossum C3	*Monodelphis domestica*	XP_001378723
RnC3	Rat C3	*Rattus norvegicus*	CAA36716
MmC3	Mouse C3	*Mus musculus*	P01027
GgC4	Chicken C4	*Gallus gallus*	T28153
CcC4-1	Carp C4-1	*Cyprinus carpio*	BAB03284
XeC4	Xenopus C4	*Xenopus laevi*	BAA11188
MmC4	Mouse C4	*Mus musculus*	P01029
HsC4A	Human C4A	*Homo sapiens*	AAB59537
HsC4B	Human C4B	*Homo sapiens*	AAA99717
CcC5-2	Carp C5-2	*Cyprinus carpio*	BAC23058
MmC5	Mouse C5	*Mus musculus*	P06684
HsC5	Human C5	*Homo sapiens*	P01031
LA2M	Horseshoe crab alpha-2-macroglobulin	*Limulus sp*	BAA19844
LjA2M	Lamprey alpha-2-macroglobulin	*Lethenteron japonicum*	BAA02762
GgOvo	Chicken ovostatin	*Gallus gallus*	P20740
HsPZP	Human pregnancy zone protein	*Homo sapiens*	X54380
HsA2M	Human alpha-2-macroglobulin	*Homo sapiens*	P01023
RnA2M	Rat alpha-2-macroglobulin	*Rattus norvegicus*	P06238
MmA2M	Mouse alpha-2-macroglobulin	*Mus musculus*	Q61838
CiA2M	Ciona alpha-2-macroglobulin	*Ciona intestinales*	NP_001027688
CcA2M	Carp alpha-2-macroglobulin1	*Cyprinus carpio*	BAA85038
XeA2M	Xenopus endodermin (alpha-2-macroglobulin-like paralog)	*Xenopus laevis*	AAB51432
CfA2M	Scallop alpha-2-macroglobulin	*Chlamys farreri*	AAR39412
OmA2M	Soft tick alpha-2-macroglobulin	*Ornithodoros moubata*	AAN10129
SsA2M	Mud crab alpha-2-macroglobulin	*Scylla serrata*	ABD61456
HsCD109s	Human CD109s	*Homo sapiens*	AAN78483

The third protein (bands 2 and 4, Figure 5) identified in the coimmunoprecipitated extracts is an alpha-amylase-like protein. The seven peptides obtained by mass spectrometry analysis matched with 2 ESTs (gi|146763124, gi|163957465). These contiged sequences display a high similarity to the alpha-amylase from the disk abalone *Haliotis discus discus* (E-value 2e-45). As alpha-amylase was known to be located mainly in the digestive tract of molluscs [58], the presence of this digestive enzyme in this context is surprising. Recovery of alpha-amylase in snail plasma is probably linked to a contamination of hemolymph by digestive mucus [32]. As it was demonstrated that porcine pancreatic alpha-amylase is able to bind N-linked oligosaccharides of glycoproteins [59], the interaction of alpha-amylase with *Sm*PoMucs or other partners of the complex could be an artefact.

There were no differences between C and IC strains in the co-immunoprecipitation experiments.

## Discussion

Two main types of immune receptor systems were described in vertebrates. Firstly, immune receptors participating to innate immune mechanisms that are encoded by germline single or multigene copy genes. And secondly, immune receptors (immunoglobulins and T cell receptors) mediating adaptive immunity that are encoded by complex multigene systems submitted to somatic rearrangement and extensive diversification processes. Immunoglobulins and T cell receptors have not been identified either in jawless vertebrates, or in deuterostome or protostome invertebrates [60] and immunity against parasites by these organisms was believed to rely exclusively on invariable germline-encoded receptors and effectors molecules that recognize antigens with low specificity. However these organisms are confronted to an environment filled with complex changing populations of microorganisms and potential pathogens, the selective pressures to which they are submitted are comparable with those of jawed vertebrates [47]. Therefore, it should be expected that they also possess sophisticated recognition systems to deal with these challenges. Recent studies support this view. In jawless vertebrate leucine rich repeat receptors genes were identified [61]. They encode a repertoire of somatically diversified receptors analogous to that of T cell Receptors or Immunoglobulins of gnathostomes and fully able to participate in an immune response [62]. For invertebrates many multigene families have been identified following immunization or examination of the genome of different species. They belong to LRR superfamily [63], [64], IgSF (Immunoglobulin SuperFamily, [17], [65]) or yet poorly characterized novel families [13], [66]. They can be integral membrane proteins, soluble, or intracellular. In invertebrates some cases of somatic adaptation have been reported for the FREPs in Molluscs [17] and for DSCAMs in arthropods [15]. In most case their involvement in immunity is not totally clarified and the interaction of these putative immune receptors with antigenic variants was never demonstrated. We started to investigate this question in the present study.

The experimental model we have chosen to answer this question is the interaction between *B. glabrata* and *S. mansoni*. As mentioned above somatically diversified immune receptors were discovered in *B. glabrata* [17] that bind to determinants of the digenetic trematode *Echinostoma paraensei*. In another trematode, *S. mansoni*, polymorphic mucins [26] called *Sm*PoMuc (for *S. mansoni* Polymorphic Mucins) displayed a high level of inter-individual polymorphism [34] and we showed that their polymorphism is the result of a complex hierarchical system (recombination, gene conversion, alternative/aberrant/trans splicing) that efficiently generates the variants based from a relatively low number of genes [27]. We suggest that these mucins could be the ligand of FREPs from *B. glabrata* [27]. In order to investigate the putative interaction between these molecules we developed a two step-experimental approach.

The first step was aimed at the identification of all the proteins from host plasma extracts that could interact with the parasite. Concerning proteins implicated in recognition and presumably in the immunity, several host lectins and parasite glycoproteins were identified. As expected, FREPs were identified as well as a novel *B. glabrata* lectin. This latter molecule displays similarities with a secreted galactose binding lectin characterised in another gastropod, *Helix pomatia* [67]. Considering the parasite molecular determinants that could be recognized by these lectins, several glycosylated proteins have been identified (Table 1). In addition to *Sm*PoMucs, two other glycoproteins were revealed in our approach: the 23 kDa integral membrane protein (Sm23) (or tetraspanin) and the glycoprotein K5. The tetraspanin was precipitated in both conditions (C/P and IC/P, Figure 1 and Table 1). The tetraspanin family includes proteins that are involved in physiological processes as diverse as egg-sperm fusion, immunological responses (antigen presentation), tissue differentiation and regulation of protein trafficking [68], [69]. In *Schistosoma mansoni* tetraspanin were studied particularly for their potential antigenic properties [41], [70], [71]. The glycoprotein K5 was identified solely in IC strain. It was known that glycoprotein K5 was encoded by a single copy gene in *S. mansoni* [42]. Four N-glycosylation sites and one signal peptide were predicted [42] and it was identified in excretory/secretory products of *S. mansoni* [72]. All these results taken together suggest that the recognition process between *S. mansoni* and *B. glabrata* could be multifactorial involving different immune receptors from the host and different carbohydrate components and/or glycoproteins from the parasite.

Host immunity relevant molecules were also revealed by this first interactome approach. Firstly, we identified a putative cytolytic protein related to β pore forming toxin family whose amino acid sequence displays significant similarities to aerolysin sequence of the bacteria *Aeromonas hydrophila* (data not shown). Aerolysins have cytolytic activity triggered by channel formation in target cell membranes. Secreted as an inactive proenzyme form from bacteria, proaerolysin binds with high affinity to the glycosyl anchor of glycosylphosphatidyl-inositol anchored proteins located on the surface membrane of target eukaryotic cells. Its binding to receptor induces a proteolytic cleavage leading to an active form that oligomerizes, forming a channel that causes lysis of the target cell. For the first time identified in a mollusc, the proteins sharing this specific pore forming sequence motif have been identified mainly in bacteria but also in a few plants and cnidarians [73], [74], [75]. In cnidarians, the pore-forming toxin could be either a defensive or offensive allomone that is involved in protecting cnidarians against predators or in killing preys [75]. In our model, aerolysin could be involved in snail innate defense responses after trematode infections.

Several other proteins that could be involved in immune processes were also identified. Some of them could be involved in molecular adhesion processes. They correspond to Dec-1-like and Matrilin-like molecules from *B. glabrata* that are suspected to be involved in extracellular matrix structure or coagulation processes [35], [76]. A peroxinectin was also identified. This cell adhesion molecule was discovered in other invertebrates species and was involved in cell attachment and spreading, nodule formation, encapsulation, agglutination and phagocytosis [77]. Two other host immune relevant molecules were precipitated: AIF (Allograft Inflammatory Factor) which was shown to be crucial in pro-inflammatory activity in innate immunity [78] and a cysteine protease inhibitor (Cystatin B, [79]). The putative functions of these different molecules are very interesting in the context of host-parasite interactions. However their suspected roles are deduced from sequence similarities and further investigations are needed to clarify their function.

Finally several other proteins were identified in the interactome approach. Their presence is worth mentioning but their role in the host/parasite interplay context remains unknown. This is the case for several Heat Shock Proteins (HSP) as well as for 3 proteins belonging to the EF-hand calcium binding family, all from *S. mansoni*. It is the case also for six parasite molecules putatively involved in the detoxification of oxidative stress [29], [80], or an anti-inflammatory, immunomodulatory protein of *S. mansoni*, *Sm*SPO-1 [81].

The second approach developed during this study was dedicated to the identification of the suspected interaction between FREPs and *Sm*PoMucs. It consisted in CoIP experiments developed with antibodies raised against *Sm*PoMucs. The FREPs and *Sm*PoMucs were found together in one molecular complex containing in addition at least a third partner, the C-terminal moiety of the ThioEster containing Protein (TEP) from *B. glabrata*. The presence of the C-terminal part of TEP in the complex is exciting as some molecules of this family were recently shown to play key roles in other invertebrate/pathogen interactions, especially in insects. Indeed, TEP1 was shown to play a crucial role in the phagocytosis of bacteria and killing of parasites in the mosquito *Anopheles gambiae*. TEP1 from the mosquito is secreted by hemocytes and cleaved in hemolymph. The C-terminal part of TEP1 binds to bacteria or ookinetes surfaces through a thioester bound. The involvement of this complement-like molecule in the antiparasitic defense of mosquitoes was recently discussed [82]. In addition, recent work demonstrates that polymorphisms in the gene encoding TEP1 occurs and explains the differences of susceptibility to *P. falciparum* between *A. gambiae* individuals [83], [84].

The identification of these three partners is very interesting in our study context. Two of them (*Sm*PoMucs and FREPs) are known to be highly variable and can display individual repertoires (see [17], [27] for details). Since the work on FREPs cited previously (Zhang et al. 2004) was performed on FREP3, we investigated the polymorphism of the FREP2 molecules specifically identified in the present study and we confirmed its high level of variability.

In principle the molecular diversity of both partners (FREPs and *Sm*PoMucs) is perfectly in agreement with their involvement in an immune complex involving several kinds of paratopes and epitopes. Future work will be developed to characterise the FREP binding site and *Sm*PoMuc molecular epitopes involved in this complex. The third partner is the TEP from *B. glabrata* (BgTEP). Precursor and phylogenetic analysis suggests that BgTEP shares the features of invertebrate TEPs that are known to be involved in antiparasitic defense and microbe phagocytosis [54], [55], [85], [86]. In addition, our LC-MS/MS experiments led to the identification of peptides that are all located in the C-terminal part of BgTEP. This suggests that BgTEP has been submitted to cleavage before its association to the two other partners of the complex. This cleavage was described for numerous members of the TEP family during the activation process, especially for TEP1 from the mosquito [52]. Therefore the BgTEP found in the complex is activated and could play a role in opsonisation processes as described for the members of this family. This hypothesis is clearly supported by the Alpha2 Macroglobulin receptor binding domain (region 1343–1427) found in the C-terminal part of BgTEP precursor. Indeed, this domain is known to be involved in the interaction with macrophage and phagocyte specific receptors [87]. A protein displaying a 18 residues N-terminal sequence identical to our BgTEP was previously characterized from *B. glabrata* [88]. It displays an α-macroglobulin proteinase inhibitor-like activity. Nevertheless, our phylogenetic analysis and the cystein array identified in the C-terminus part of the Bg TEP [51], [52], [54] strongly support that BgTEP belongs to the invertebrate TEP and not to the A2M group.

As FREPs display a high level of similarity among themselves, it is difficult to identify without doubt the isoform(s) present in the immune complex characterised by mass spectrometry. Nevertheless, we identify a FREP2-specific peptide and consequently, we are sure that FREP2 is present in the immune complex, alone or in combination with other FREPs. This result is interesting because *FREP2* is the main gene of the *FREP* family up-regulated following exposure to *S. mansoni* [25], [46], [89]. Moreover, our analysis of BgBRA-FREP2 diversity in the present study reveals that somatic processes probably occurs and increase their repertoire in individuals. Consequently, FREP2 could represent a sort of induced or selected “antibody” following parasite infection and dedicated to parasite determinant recognition.

Finally, the results obtained in this work could help understanding different results obtained during previous population studies. These studies of the interaction between *B. glabrata* and *S. mansoni* have revealed a phenomenon called compatibility polymorphism [90]. In natural populations, some snail/schistosome combinations are compatible and others are not, the success or the failure of *B. glabrata*/*S. mansoni* infection depending on the matched or mismatched status of the host and parasite phenotypes [90]. The molecular basis of this phenomenon is unknown but molecular determinants like those revealed through this study are promising candidates. Indeed, we can hypothesize that particular combinations of FREPs and *Sm*PoMucs expressed by individuals could interact together or not to define the matched or mismatched status evoked previously. We have recently shown that each *S. mansoni* individual expresses a particular *Sm*PoMuc profile [27] that could be recognized or not by a particular FREPs profile expressed by the infected mollusc. We are currently testing this hypothesis by analysing the concordance of alleles in compatible combinations in different populations of *B. glabrata* and *S. mansoni* in interaction. If this hypothesis is verified, it could illustrate a bet hedging strategy of the parasite based on a diversification/polymorphism process providing an opportunity to certain individuals to infest a host permitting parasite species perpetuation. Bet hedging strategies are well characterized in bacteria [91] and consists in a switching between phenotypes for species confronted to fluctuating and unpredictable environmental variations. The FREP somatic diversification of mollusc individuals is insufficient to allow recognition of all parasite individuals. This somatic diversification could represent a first step towards adaptive immunity in an invertebrate species: individuals are capable of somatic diversification of their immune receptors allowing for an enlargement of their recognition capacity, nevertheless, this repertoire is smaller than the vertebrate immune receptor repertoire and does not allow for the recognition of all putative antigens entering in contact with a given individual. In the future an analysis of the germ line genes of FREP that will allow dn/ds calculations and the modelling of the FREP domains bound to their mucin ligand once crystals are available should shed light on the properties of these variants and on their necessity.
